# Concurrent Mold, Mycobacterial, and Viral Infections in a Hematopoietic Stem Cell Transplant Recipient Undergoing Lung Transplantation for Graft-Versus-Host Disease

**DOI:** 10.3390/curroncol33030145

**Published:** 2026-03-02

**Authors:** Layan Akkielah, Wayne Leung, Serena Wang, Lili Ataie, Anargyros Xenocostas, Asma Syed, Ying-Han R. Hsu, Michael Silverman, Fatimah AlMutawa, MohammadReza Rahimi Shahmirzadi

**Affiliations:** 1 Department of Medicine, Division of Infectious Diseases, Western University, London Health Sciences Centre, London, ON N6A 4V2, Canada; 2 Division of Hematology, Department of Medicine, Western University, London Health Sciences—Victoria Hospital, London, ON N6A 4V2, Canada; 3 Department of Transplant Infectious Diseases, Division of Infectious Diseases, University Health Network, University of Toronto, Toronto, ON M5G 2C4, Canada; 4 Laboratory Medicine Program, University Health Network, Toronto, ON M5G 2C4, Canada; 5 Department of Pathology and Laboratory Medicine, Schulich School of Medicine & Dentistry, Western University, London, ON N6A 4V2, Canada

**Keywords:** hematopoietic stem cell transplantation, non-aspergillus mold infection, nontuberculous mycobacteria

## Abstract

Hematopoietic stem cell transplant (HSCT) recipients are at high risk for opportunistic infections due to profound immunosuppression and graft-versus-host disease (GvHD). Molds and nontuberculous mycobacteria (NTM) pose diagnostic and therapeutic challenges, especially when infections overlap. A 42-year-old woman with prior allogeneic HSCT for acute myeloid leukemia (AML) developed pulmonary infections with *Microascus* spp. and *Mycobacterium chimaera*, later complicated by *Aspergillus calidoustus* and RSV infection. Initial therapy included voriconazole, amphotericin B, and a macrolide-based multidrug regimen for NTM. Modifications were required for drug resistance and hepatotoxicity. Despite partial response, recurrent fungal infection necessitated prolonged antifungal therapy, including adjunctive inhaled amphotericin B and terbinafine. Ultimately, progressive bronchiolitis obliterans prompted bilateral lung transplantation. Explant pathology revealed necrotizing granulomas positive for NTM and *Microascus* spp. Post-transplant prophylaxis with voriconazole, rifabutin, azithromycin, and inhaled amikacin prevented recurrence, and the patient remained clinically stable at 6-month follow-up. This case illustrates the complexity of managing overlapping mold and NTM infections in HSCT recipients, highlighting the need for individualized, multidisciplinary care. Therapeutic drug monitoring, careful adjustment for drug–drug interactions, and the use of adjunctive inhaled antifungals were critical to achieving a favorable outcome.

## Simple Summary

Patients who undergo stem cell transplantation for blood cancers often develop long-term immune system problems that increase their risk of serious infections. Some infections are difficult to diagnose and treat, especially when more than one infection occurs at the same time. We describe a woman who developed multiple rare lung infections years after her stem cell transplant, including fungal and mycobacterial infections, followed by respiratory viral infection. Treating these infections was challenging because of medication side effects, drug interactions, and limited treatment guidance. Despite prolonged therapy, progressive lung damage ultimately required lung transplantation. Examination of the removed lungs confirmed evidence of prior treated infections. With careful long-term antimicrobial prevention and close monitoring, she recovered well after transplantation. This case highlights the complexity of managing overlapping infections in severely immunocompromised patients and emphasizes the importance of individualized, multidisciplinary care and careful medication management.

## 1. Introduction

In the setting of profound immunosuppression, HSCT recipients are prone to serious infections, including invasive fungal infections, Aspergillus and non-Aspergillus mold infections (NAMIs) and NTM infections, especially when GVHD occurs [1]. While these infections are recognized to be complicated in the literature, their simultaneous occurrence in the context of post-HSCT is rarely reported. We present a case of a 42-year-old woman post-HSCT for AML who developed concurrent pulmonary infections with *Microascus* spp., *Mycobacterium chimaera*, and RSV followed by *Aspergillus calidoustus*. This case highlights the complexities of diagnosing and managing rare opportunistic infections in immunocompromised hosts, emphasizing drug/drug interactions, therapeutic drug monitoring, and antimicrobial resistance. We aim to contribute to the growing body of literature on the co-management of NAMIs, NTM and RSV in HSCT recipients.

## 2. Case Report

**Figure 1:**
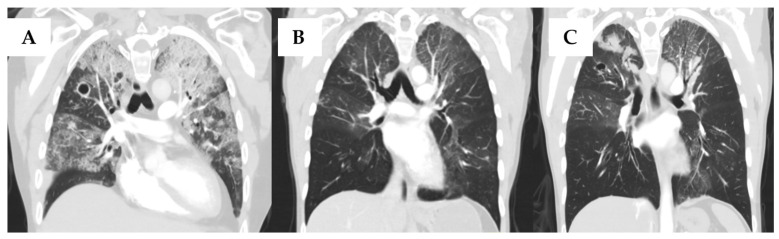
Computed tomographic (CT) scans of the lung of a 42-year-old female with nontuberculous mycobacterial infection (NTM) and non-*Aspergillus* invasive mold infection (NAMI) in a hematopoietic cell transplant recipient, who subsequently received bilateral lung transplantation. (**A**) Multifocal ground-glass opacities more prominent in the left lung; 1.5 cm cavitary lesion in the right upper lobe with centrilobular nodularity reported prior to NTM and NAMI therapy initiation. (**B**) Near-complete clearing of severe bilateral airspace consolidations after treatment of *Microascus* spp. and *Mycobacterium chimaera* infections. (**C**) Bilateral consolidative changes in the upper lobes, mostly at the time of diagnosis of *Aspergillus calidoustus* infection.

A 42-year-old woman underwent a 10/10 HLA-matched sibling peripheral blood stem-cell transplant for FLT3-ITD-positive acute myeloid leukemia after first achieving complete remission with 7 + 3 induction and one cycle of high-dose cytarabine consolidation. Conditioning was myeloablative busulfan and cyclophosphamide. On post-stem cell transplant day (PSCTD) day 134, she developed moderate chronic GVHD (cGVHD) of the mouth, skin, liver and eyes. Over the following year, this evolved into severe pulmonary cGVHD and, over time, pulmonary chronic graft-versus-host disease progressed, with the development of bronchiolitis obliterans that ultimately evolved into severe end-stage lung disease, necessitating therapy with prednisone at 1 mg/kg, mycophenolate mofetil, azithromycin, umeclidinium/vilanterol and montelukast. Her infectious prophylaxis regimen included acyclovir 400 mg twice daily, penicillin V 300 mg twice daily, posaconazole 300 mg delayed-release daily, and trimethropirm-sulfamethoxezaole (one double-strength tablet three times weekly). Five years after HSCT, she experienced worsening respiratory symptoms and the development of cavitary right upper-lobe lesions warranting serial bronchoscopies that yielded *Mycobacterium chimaera* together with *Microascus* spps. and a positive serum galactomannan. Antimicrobial therapy was initiated, which included 12 weeks of intravenous amikacin, azithromycin, and ethambutol, which was later substituted with Moxifloxacin due to her underlying eye GVHD) and rifampicin, followed by clofazimine and 12 months of rifabutin, substituted due to liver toxicity and *Mycobacterium chimaera* resistance pattern. The microascus infection treatment regimen included 12 weeks of voriconazole, and inhaled and intravenous liposomal amphotericin B for *Microasuas* species. Management was repeatedly interrupted due to adverse events including hepatotoxicity with rifampin, visual concerns with ethambutol and pronounced QTc prolongation to 628 ms, necessitating temporary cessation of clofazimine and rifabutin. Near-complete clinical and radiological resolution was reported on repeated imaging 3 months after extensive treatment, with subsequent bronchoalveolar lavage samples negative for NTM (Figure 1).

The following year, she developed new bilateral pulmonary consolidations, and bronchoalveolar lavage cultures grew a non-fumigatus *Aspergillus* species, representing *Aspergillus calidoustus* (Table 1). She was treated with isavuconazole, which was discontinued due to skin rash and itching, and therapy was transitioned to liposomal amphotericin B in combination with terbinafine. She completed 12 weeks of systemic therapy before transitioning to inhaled amphotericin B. For her RSV infection, she received 10 days of Ribavirin given her immunosuppressed state and the presence of lower respiratory tract infection.

**Figure 2:**
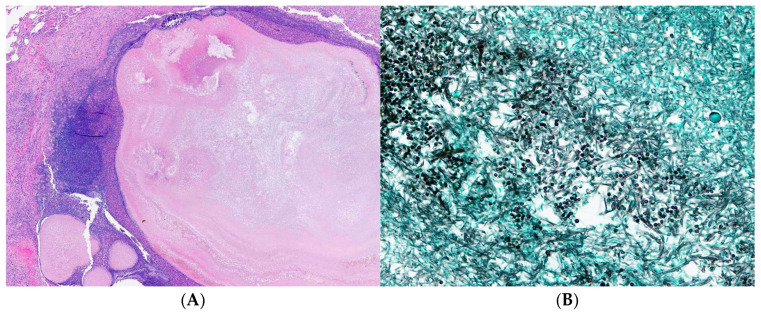
(**A**) Necrotizing granuloma associated with mold infection (H&E; original magnification: 20×); (**B**) the fungal elements consisted of hyaline septate hyphae, conidia and asci (Grocott methenamine silver stain; original magnification: 400×). Fungal polymerase chain reaction performed on DNA extracted from formalin-fixed paraffin-embedded tissue targeting the internal transcribed spacer region of ribosomal RNA genes identified the fungi as *Microascus* species.

**Figure 3:**
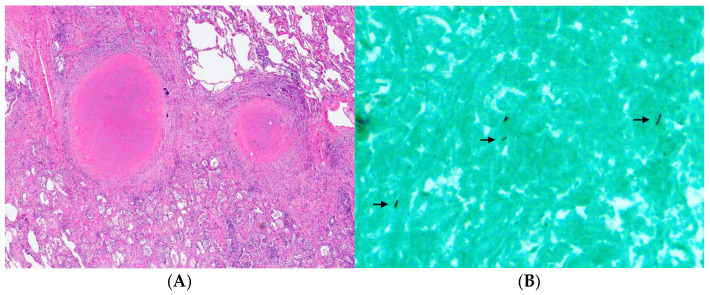
(**A**) Small necrotizing granulomata with fine dystrophic calcification (H&E; original magnification: 20×). (**B**) A few bacilli were visualized by Grocott methenamine silver stain (arrows; original magnification: 400×) without uptake of Ziehl–Neelsen stain, suggesting nonviable nontuberculous mycobacteria.

Despite these interventions, she developed progressive bronchiolitis obliterans related to pulmonary cGVHD and ultimately underwent bilateral lung transplantation at a regional lung transplant program; intraoperative veno-arterial ECMO was used, and she was discharged on postoperative day 12. Explant histopathology demonstrated multiple partly calcified necrotizing granulomas consistent with treated pulmonary aspergillosis, and nontuberculous mycobacterial infection, with PCR detection of *Microascus* spp. (Figure 2 and Figure 3).

Post-transplant prophylaxis included voriconazole, azithromycin, rifabutin, and inhaled amikacin, in addition to trimethoprim-sulfamethoxazole and valganciclovir, given the antecedent invasive mold disease and prior NTM infection. Bronchoscopies post lung transplant were negative for NTM and *Aspergillus* spp. Post-transplant immunosuppression consisted of tacrolimus, mycophenolate sodium, and prednisone. The patient reported improved exercise tolerance with daily walking and light resistance training twelve months after lung transplantation, along with independence in household activities and childcare, stable pulmonary function, and no rejection on protocol assessments.

## 3. Discussion

This case illustrates the challenges of diagnosing and managing concurrent rare mold, nontuberculous mycobacterial (NTM), and RSV in a patient with immune dysfunction following an allogenic hematopoietic stem cell transplant (HSCT). The risk of acquiring opportunistic infections in HSCT recipients is substantial due to prolonged and multifactorial immune dysfunction involving innate, humoral, and cellular immunity, with infectious complications remaining a leading cause of morbidity and mortality [1,2,3,4]. Recipients with chronic graft-versus-host disease (cGVHD) are at an amplified risk, particularly when pulmonary involvement requires prolonged corticosteroid use and additional immunosuppressive agents [5,6,7]. Pulmonary cGVHD leads to structural lung damage, impaired mucociliary clearance, and chronic inflammation, creating an environment that favors persistent and invasive infections [5,6,7].

Although uncommon, NTM infections are increasingly recognized in HSCT recipients, particularly among patients with cGVHD and underlying structural lung abnormalities [8,9,10]. Pulmonary NTM disease is the most frequent presentation and typically appears as nodular, cavitary, or bronchiectatic changes on imaging [9,10,11]. A key clinical challenge is distinguishing true NTM disease from colonization in immunocompromised hosts, where chronic respiratory symptoms and persistent radiographic abnormalities may occur independent of active infection [9,10,11].

*Mycobacterium chimaera*, which is a slow-growing member of the *Mycobacterium avium* complex, has been increasingly reported among immunocompromised hosts, including HSCT recipients, and has been described as a predominant species within this population [10,11,12]. Initially recognized in association with the heater–cooler units used during cardiac surgery, M. chimaera is now known to cause chronic pulmonary and intrathoracic disease in immunocompromised patients, with reported mortality rates exceeding 20% [12].

Current guidance for NTM pulmonary disease recommends susceptibility-guided multidrug therapy administered for prolonged durations (typically ≥12 months after culture conversion), but the HSCT-specific evidence base remains limited and much of practice is extrapolated from broader populations [12,13].

In this patient, multidrug NTM therapy required repeated modification due to hepatotoxicity, QTc prolongation, and other adverse effects, limitations that are well-recognized barriers to successful NTM treatment in practice. The inclusion of inhaled liposomal amikacin as part of a refractory strategy is supported by evidence demonstrating a benefit in treatment-refractory MAC lung disease, and is consistent with guideline-supported escalation approaches in difficult-to-treat NTM pulmonary infection [13,14].

Simultaneously, this case underscores the challenges of non-*Aspergillus* mold infections (NAMIs) in profoundly immunocompromised hosts, particularly in the setting of chronic graft-versus-host disease. Non-*Aspergillus* molds are ubiquitous environmental organisms typically acquired through inhalation. Although NAMIs remain relatively uncommon in hematopoietic stem cell transplant recipients, they are associated with high mortality rates, limited antifungal susceptibility breakpoints, significant diagnostic uncertainty, and an incompletely defined cumulative incidence [15,16,17]. The most frequently reported NAMIs in this population include the *Mucorales* and *Fusarium* species; however, an increasing number of emerging molds, such as the *Scopulariopsis* and *Microascus* species, are being recognized [18,19,20,21]. In the present case, explant lung histopathology demonstrated necrotizing granulomas with PCR detection of *Microascus* spp., confirming true invasive non-*Aspergillus* mold infection rather than colonization. This finding illustrates the limitations of histopathology alone, as *Microascus* species may be morphologically indistinguishable from other filamentous fungi, including *Aspergillus* and *Fusarium*, and underscores the importance of molecular diagnostic techniques in establishing a definitive diagnosis in immunocompromised patients [22,23].

The management of NAMIs requires an individualized, multidisciplinary approach guided by accurate organism identification, antifungal susceptibility data when available, and careful consideration of drug–drug interactions and cumulative toxicity. The treatment of *Microascus* infections is particularly challenging due to the absence of established susceptibility breakpoints, limited clinical data, and consistently high minimum inhibitory concentrations to multiple antifungal agents, including itraconazole and amphotericin B, often necessitating tailored combination strategies despite limited clinical evidence [17,18,23]. Accordingly, consensus guidance supports a case-by-case approach, with lipid formulations of amphotericin B, voriconazole, and combination antifungal therapy considered reasonable options in selected patients. In this patient, prolonged therapy with liposomal amphotericin B combined with terbinafine, followed by adjunctive inhaled amphotericin B, was employed in the context of limited susceptibility data, cumulative drug toxicity, and progressive pulmonary disease, aligning with available expert recommendations [16,17,19,22,24,25,26]. Definitive management emphasizes both the reversal of immunodeficiency and surgical source control. In this case, bilateral lung transplantation provided definitive source control by removing structurally damaged lung tissue; however, it did not reverse the underlying immune dysfunction. This likely contributed to the absence of recurrent invasive mold infection on follow-up.

The identification of azole-resistant *Aspergillus calidoustus* (section Usti) introduced additional therapeutic constraints in this case, as this species is increasingly recognized as a clinically relevant pathogen in immunocompromised hosts and is frequently associated with reduced susceptibility to triazole antifungals, often necessitating alternative treatment strategies [27]. In such settings, amphotericin B-based regimens and combination antifungal therapy are commonly required, particularly when azole resistance or intolerance limits therapeutic options, consistent with expert guidance for the management of non-Aspergillus mold infections [16,18].

Despite prolonged and aggressive antimicrobial therapy, the patient ultimately required bilateral lung transplantation for progressive bronchiolitis obliterans secondary to pulmonary chronic graft-versus-host disease, a recognized late complication of allogeneic HSCT [5,6,7]. The presence of necrotizing granulomas with evidence of both nontuberculous mycobacterial and mold infection on explant pathology supports the interpretation of treated or partially treated infection at the time of transplantation and underscores the importance of careful post-transplant antimicrobial suppression or secondary prophylaxis in high-risk patients [5,6,7,15]. In patients with antecedent invasive mold disease, secondary antifungal prophylaxis has been shown to reduce the risk of recurrence following allogeneic HSCT; accordingly, voriconazole secondary prophylaxis was selected in this case based on the available clinical data [28,29].

The patient’s clinical course was further complicated by respiratory syncytial virus (RSV) infection, an important cause of lower respiratory tract disease in HSCT recipients, in whom progression from upper to lower tract infection is associated with increased morbidity and mortality [30,31]. Although vaccines and monoclonal antibodies have expanded preventive options in selected populations, their role in HSCT recipients remains incompletely defined, and management in this group continues to rely largely on observational data and transplant-focused reviews [32,33].

Overall, this case highlights the need for the individualized, multidisciplinary management of HSCT recipients with overlapping opportunistic infections, including frequent reassessment of microbiologic data to distinguish colonization from invasive disease, proactive mitigation of drug–drug interactions and cumulative toxicities, and the use of therapeutic drug monitoring to optimize antimicrobial exposure [13,16]. The judicious use of adjunctive inhaled antifungal and antimycobacterial therapies, in conjunction with tailored systemic treatment, contributed to clinical stabilization and ultimately enabled lung transplantation in a patient with otherwise progressive pulmonary chronic graft-versus-host disease [16,24,25].

## 4. Conclusions

This case highlights the complexity of managing overlapping mold, nontuberculous mycobacterial, and viral infections in a patient with chronic immune dysfunction following hematopoietic stem cell transplantation. Prolonged immunosuppression, pulmonary chronic graft-versus-host disease, structural lung damage, and cumulative antimicrobial toxicities created significant diagnostic and therapeutic challenges. Successful management required multidisciplinary collaboration, repeated reassessment of microbiologic data to distinguish colonization from invasive disease, therapeutic drug monitoring, and careful mitigation of drug–drug interactions. Ultimately, bilateral lung transplantation provided definitive source control in the setting of progressive bronchiolitis obliterans, with tailored post-transplant antimicrobial suppression preventing recurrence. This case underscores the need for individualized treatment strategies, the integration of molecular diagnostics, and close coordination between infectious diseases, transplant, and hematology teams when confronting rare and concurrent opportunistic infections in highly immunocompromised hosts.

## Figures and Tables

**Table 1:** Pathogens identified on bronchoalveolar lavage samples.

Pathogens	Minimum Inhibitory Concentrations (If Applicable)
*Mycobacterium chimaera*	Amikacin 16 μg/mL (susceptible) Clarithromycin 1 μg/mL (susceptible) Linezolid 16 μg/mL (intermediate) Moxifloxacin 5 μg/mL (resistant)
*Microascus* spp.	Amphotericin B 1 μg/mL (no breakpoints) Caspofungin < 0.0080 g/mL (no breakpoints) Itraconazole > 16 μg/mL (no breakpoints) Voriconazole 2 μg/mL (no breakpoints)
*Aspergillus calidoustus*	Susceptibility data were not available

## Data Availability

The original contributions presented in this study are included in the article. Further inquiries can be directed to the corresponding author.
